# Supplementary material for: Comparing a Sensor for Movement Assessment with Traditional Physiotherapeutic Assessment Methods in Patients after Knee Surgery—A Method Comparison and Reproducibility Study
Source: Int J Environ Res Public Health. 2022 Dec 9;19(24):16581. doi: 10.3390/ijerph192416581 (PMC9779175; doi:10.3390/ijerph192416581)

Supplementary Figure S1: Scatterplots of the measurements made at the injured and the contralateral leg of each patient. Observations from both rounds are included in the same plot. The red line indicates equality of the two measurements.

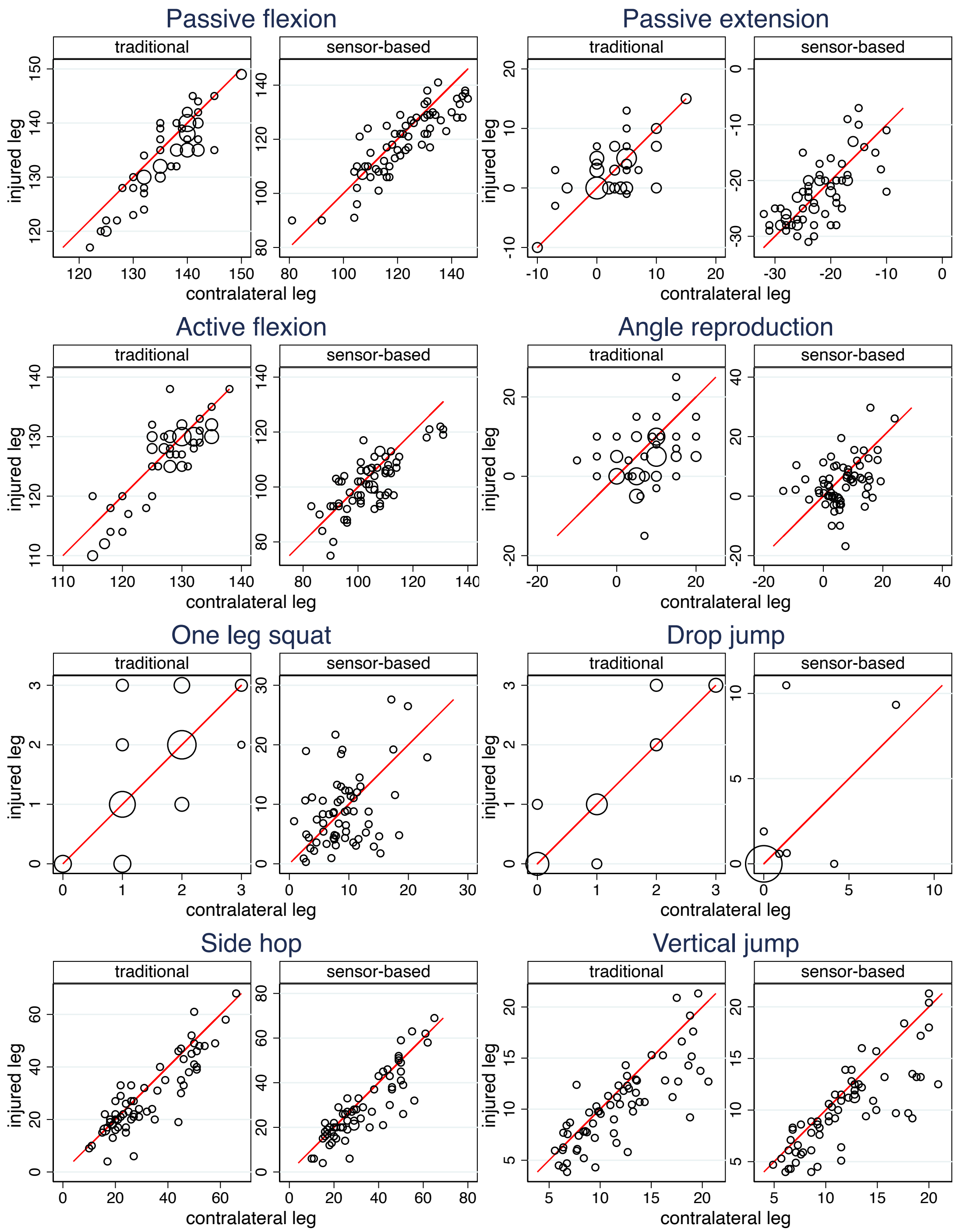

Supplement: Supplementary file 1 [file ijerph-19-16581-s001.zip › Figure S1.pdf]
