# Supplementary material for: Comparing a Sensor for Movement Assessment with Traditional Physiotherapeutic Assessment Methods in Patients after Knee Surgery—A Method Comparison and Reproducibility Study
Source: Int J Environ Res Public Health. 2022 Dec 9;19(24):16581. doi: 10.3390/ijerph192416581 (PMC9779175; doi:10.3390/ijerph192416581)
Supplement: Supplementary file 1 [file ijerph-19-16581-s001.zip › File S1.pdf]

Supplemental File S1: Verbal instructions

This file refers to the article

Comparing a sensor for movement assessment with traditional physiotherapeutic assessment methods in patients after knee surgery – a method comparison and reproducibility study

by Jennifer Eymann, Werner Vach, Luis Fischer, Marcel Jakob, and Andreas Gösele.

Vorbereitung:

Die nachfolgenden Tests werden alle barfuss durchgeführt. Der Testleiter informiert sie über Start und Ende der Messung. Während des Countdowns muss das Bein immer so ruhig wie möglich gehalten werden.

Passive Winkelmessung

Setzen Sie sich auf die Liege und strecken Sie die Füße aus. Halten Sie das Bein während des Countdowns ruhig. Ziehen Sie das Knie mit dem Sensor soweit wie möglich an, in dem Sie mit Ihren Händen hinter die Kniekehle greifen. Schieben Sie dieses Bein wieder in die Ausgangsposition zurück. Halten Sie mit der Ferse während des Tests unbedingt den Bodenkontakt.

Streckdefizit

Setzen Sie sich mit geradem Rücken auf die Erhöhung. Das nicht getestete Bein ist ca. 40° angewinkelt. Die Rolle wird von der Testleiterin unter Ihrer Kniekehle platziert. Halten Sie das Bein während des Countdowns ruhig. Die Testleiterin entfernt die Rolle. Lassen Sie Ihre Kniekehle zum Boden sinken, soweit es das Bein erlaubt. Halten Sie mit der Ferse während dem Test unbedingt den Bodenkontakt.

One Leg Sqaut

Stellen Sie sich auf die Erhöhung. Die Arme stützen Sie in der Hüfte ein. Halten Sie das Bein während des Countdowns ruhig. Gehen Sie so weit in die Beugung, bis Sie mit der Ferse des anderen Beins den Boden berühren. Gehen Sie danach wieder in die Ausgangsposition.

Drop Jump

Stellen Sie sich mit beiden Beinen auf die Erhöhung. Stemmen Sie die Hände in die Hüfte. Halten Sie das Bein während des Countdowns ruhig. Springen Sie beidbeinig nach vorne auf den Boden und dann sofort wieder nach oben. Landen Sie wieder mit beiden Beinen.

Aktive Winkelmessung

Stellen Sie sich aufrecht vor den Stuhl. Ihre Oberschenkel berühren während des gesamten Tests den Stuhl. Halten Sie das Bein während des Countdowns ruhig. Winkeln Sie das Bein mit Sensor so weit wie möglich an und stellen sich wieder in die Ausgangsposition. Bewegen Sie den Oberschenkel nicht nach vorne oder nach hinten. Die Knie müssen parallel nebeneinander bleiben. Führen Sie den Test langsam und ohne Schwung durch.

Winkelreproduktion

Stellen Sie sich aufrecht vor den Stuhl hin. Ihre Oberschenkel berühren den Stuhl. Halten Sie das Bein während des Countdowns ruhig. Ein Winkel wird Ihnen vorgegeben. Winkeln Sie das Bein mit Sensor an, bis Sie den angegebenen Winkel erreichen. Verharren Sie in dieser Position, solange die Testleiterin sie dazu auffordert. Gehen Sie zwei Schritte und kehren in die Ausgangsposition zurück. Winkeln Sie das Bein auf Anweisung der Testleiterin erneut an, ohne dass Sie den Winkel auf dem Display sehen. Versuchen Sie nun denselben Winkel zu erreichen, der Ihnen vorher angegeben wurde. Verharren Sie in dieser Position, solange die Testleiterin sie dazu auffordert. Der Test wird drei Mal wiederholt.

Vertical Jump

Stellen Sie sich aufrecht hin. Stemmen Sie die Hände in die Hüften. Halten Sie das Bein während des Countdowns ruhig. Winkeln Sie das Bein ohne Sensor an. Dieses darf den Boden nicht berühren. Springen Sie mit dem Bein am Sensor maximal hoch. Landen Sie wieder auf diesem einen Bein. Das Sprungbein wird in der Luft gestreckt

Side Hop

Stellen Sie sich aufrecht an die Linie. Halten Sie das Bein während des Countdowns ruhig. Springen Sie für 30 Sek. von links nach rechts über die Linien.
