# Supplementary material for: Comparing a Sensor for Movement Assessment with Traditional Physiotherapeutic Assessment Methods in Patients after Knee Surgery—A Method Comparison and Reproducibility Study
Source: Int J Environ Res Public Health. 2022 Dec 9;19(24):16581. doi: 10.3390/ijerph192416581 (PMC9779175; doi:10.3390/ijerph192416581)
Supplement: Supplementary file 1 [file ijerph-19-16581-s001.zip › Table S1.pdf]

Supplementary Table S1: Further results on the reproducibility

Mean difference:

	Raw measurements		Side differences	
	Traditional assessment	Sensor-based assessment	Traditional assessment	Sensor-based assessment
Passive flexion	0.08	-0.09	0.03	-0.34
Passive extension	0.05	-1.21	-0.39	-0.36
Active flexion	-0.06	-0.38	-0.91	-1.30
Angle reproduction	-0.45	0.05	-1.45	-2.97
One leg squat	-0.05	-0.62	0.10	1.54
Drop jump	-0.09	-0.32	0.06	-0.55
Side hops	3.89	3.52	-0.61	-2.19
Vertical jump	0.40	0.41	-0.65	-0.63

Standard deviations of differences:

	Raw measurements		Side differences	
	Traditional assessment	Sensor-based assessment	Traditional assessment	Sensor-based assessment
Passive flexion	3.20	6.37	2.97	6.77
Passive extension	1.14	3.48	1.58	4.24
Active flexion	2.92	4.33	2.79	6.22
Angle reproduction	7.53	9.18	11.61	13.00
One leg squat	0.76	5.16	1.01	7.43
Drop jump	0.38	1.35	0.56	2.14
Side hops	5.93	5.65	7.16	7.33
Vertical jump	1.55	1.73	1.93	1.75

p-value:

	Raw measurements		Side differences	
	traditional assessment	Sensor-based assessment	traditional assessment	Sensor-based assessment
Passive flexion	0.848	0.907	0.954	0.776
Passive extension	0.748	0.006	0.162	0.626
Active flexion	0.867	0.480	0.070	0.237
Angle reproduction	0.625	0.962	0.477	0.199
One leg squat	0.616	0.352	0.598	0.259
Drop jump	0.184	0.178	0.668	0.301
Side hops	<0.001	<0.001	0.637	0.106

Vertical jump	0.045	0.060	0.070	0.049

Limits of agreement:

	Raw measurements		Side differences	
	traditional assessment	Sensor-based assessment	traditional assessment	Sensor-based assessment
Passive flexion	-6.19 - 6.34	-12.57 - 12.39	-5.80 - 5.86	-13.61 - 12.92
Passive extension	-2.19 - 2.29	-8.04 - 5.62	-3.49 - 2.70	-8.68 - 7.95
Active flexion	-5.78 - 5.66	-8.86 - 8.10	-6.37 - 4.56	-13.49 - 10.88
Angle reproduction	-15.21 - 14.30	-17.93 - 18.04	-24.21 - 21.30	-28.44 - 22.51
One leg squat	-1.53 - 1.43	-10.73 - 9.50	-1.89 - 2.08	-13.02 - 16.09
Drop jump	-0.83 - 0.65	-2.97 - 2.33	-1.03 - 1.15	-4.75 - 3.64
Side hops	-7.73 - 15.51	-7.56 - 14.60	-14.64 - 13.42	-16.56 - 12.17
Vertical jump	-2.64 - 3.45	-2.98 - 3.81	-4.44 - 3.13	-4.06 - 2.80

RMSE:

	Raw measurements		Side differences	
	traditional assessment	Sensor-based assessment	traditional assessment	Sensor-based assessment
Passive flexion	3.17	6.32	2.93	6.67
Passive extension	1.13	3.66	1.60	4.19
Active flexion	2.90	4.31	2.89	6.26
Angle reproduction	7.48	9.11	11.52	13.14
One leg squat	0.75	5.16	1.00	7.47
Drop jump	0.38	1.37	0.54	2.15
Side hop	7.05	6.62	7.07	7.53
Vertical jump	1.59	1.77	2.01	1.84

Mean absolute deviation:

	Raw measurements		Side differences	
	traditional assessment	Sensor-based assessment	traditional assessment	Sensor-based assessment
Passive flexion	2.23	4.62	2.15	4.97
Passive extension	0.35	2.42	0.70	3.39
Active flexion	2.09	3.26	2.30	5.12
Angle reproduction	5.61	6.86	8.18	9.53
One leg squat	0.40	3.88	0.61	5.88
Drop jump	0.15	0.41	0.29	0.82

Side hops	5.69	5.45	5.00	5.48
Vertical jump	1.14	1.15	1.65	1.27
