# Supplementary material for: Comparing a Sensor for Movement Assessment with Traditional Physiotherapeutic Assessment Methods in Patients after Knee Surgery—A Method Comparison and Reproducibility Study
Source: Int J Environ Res Public Health. 2022 Dec 9;19(24):16581. doi: 10.3390/ijerph192416581 (PMC9779175; doi:10.3390/ijerph192416581)
Supplement: Supplementary file 1 [file ijerph-19-16581-s001.zip › Table S2.pdf]

Supplementary Table S2: Further results on the agreement between the two modalities

Mean difference:

	Raw measurements	Side differences
Passive flexion	15.23	-0.83
Passive extension	25.13	-0.35
Active flexion	24.66	-1.47
Angle reproduction	0.39	0.85
Side hops	-0.23	-0.11
Vertical jump	0.10	-0.27

Standard deviation of differences:

	Raw measurements	Side differences
Passive flexion	11.78	6.64
Passive extension	6.01	4.32
Active flexion	9.03	6.42
Angle reproduction	2.99	3.46
Side hops	4.32	4.75
Vertical jump	1.03	1.60

p-value:

	Raw measurements	Side differences
Passive flexion	<0.001	0.317
Passive extension	<0.001	0.515
Active flexion	<0.001	0.067
Angle reproduction	0.137	0.050
Side hops	0.551	0.853
Vertical jump	0.301	0.183

Limits of agreement:

	Raw measurements	Side differences
Passive flexion	-7.86 - 38.32	-13.84 - 12.18
Passive extension	13.35 - 36.91	-8.82 - 8.13
Active flexion	6.95 - 42.36	-14.05 - 11.11
Angle reproduction	-5.48 - 6.26	-5.94 - 7.64
Side hops	-8.70 - 8.24	-9.41 - 9.19
Vertical jump	-1.93 - 2.12	-3.41 - 2.86

RMSE:

	Raw measurements	Side differences
Passive flexion	19.23	6.64
Passive extension	25.83	4.30
Active flexion	26.25	6.54
Angle reproduction	3.01	3.54

Side hops	4.31	4.71
Vertical jump	1.03	1.61

Mean absolute deviation:

	Raw measurements	Side differences
Passive flexion	15.72	5.29
Passive extension	25.13	3.41
Active flexion	24.66	5.32
Angle reproduction	2.09	2.82
Side hops	2.18	2.71
Vertical jump	0.69	1.01
